# Molecular characterization of *Mannheimia haemolytica* isolates associated with pneumonic cases of sheep in selected areas of Central Ethiopia

**DOI:** 10.1186/s12866-018-1338-x

**Published:** 2018-12-05

**Authors:** Abinet Legesse, Takele Abayneh, Gezahegne Mamo, Esayas Gelaye, Liyuwork Tesfaw, Martha Yami, Alebachew Belay

**Affiliations:** 1 National Veterinary Institute, P.O. Box 19, Bishoftu, Ethiopia; 2 College of Veterinary Medicine and Agriculture, Addis Ababa University, P.O. Box 34, Bishoftu, Ethiopia

**Keywords:** Central Ethiopia, *Mannheimia haemolytica*, Molecular characterization, Sheep

## Abstract

**Background:**

*Mannheimia haemolytica* has been recognized as the principal cause of pneumonic pasteurellosis in sheep and goats. It is one of the important diseases of small ruminants in Ethiopia. While annual vaccination using a monovalent vaccine (inactivated *Pasteurella multocida* biotype A) is common, respiratory diseases are still reported in various parts of Ethiopia. This suggests the need for further investigation into the species and strains responsible for the disease, which is vital information for development of a multivalent vaccine. The objective of the current study was to isolate *M. heamolytica* associated with pneumonic cases of sheep in selected areas of Central Ethiopia, determine its role and the strains/genotypes of the bacterium circulating in the study area.

**Results:**

Bacteriological analysis of nasal swab samples collected from a total of 76 pneumonic cases of sheep showed that *M. haemolytica* was isolated from 26 of them while *B.trehalosi* from two cases*.* Further molecular analyses of the isolates using *M. haemolytica* species-specific and *M.haemolytica* serotype-1 antigen specific PCR assays revealed, 26 of the isolates were identified as *M. haemolytica* of which 21 of them were *M. haemolytica* serotype-1. Both *M. haemolytica* and *B.trehalosi* isolates were not detected in a PCR assay targeting capsular biosynthesis gene (*capA*) of *P.multocida* despite the non-specific products observed in *M. haemolytica* isolates. Phylogenetic analysis of *M. haemolytica* isolates included in this study in comparison with the reference strains with respect to *PHSSA* and *Rpt2* genes revealed that the Ethiopian *M. haemolytica* isolates constituted three distinct genotypes consistent with site of origin.

**Conclusion:**

The study indicated that *M.haemolytica* is commonly associated with cases of pneumonia in sheep in the study areas of central Ethiopia although the remaining other pathogens responsible for majority of the cases are yet to be determined. Molecular characterization revealed the existence of three genotypes of *M. haemolytica* circulating in the study areas consistent to the site of isolation. The findings suggest further extensive work to determine all pathogens associated with sheep pneumonia and the strain distribution of *M. heamolytica* to understand its molecular epidemiology at national level and design cost effective prevention and control methods.

## Introduction

Sheep constitute a significant proportion of the Ethiopian livestock industry, with the total population estimated at 26.1 million [1, 2]. However, the productivity of sheep is unsatisfactory largely due to diseases and poor animal management practices [3, 4].

Diseases causing respiratory problems in sheep have been known of great economic impact in the central highlands of Ethiopia with frequent records of outbreaks and mortalities [5].

Ovine pasteurellosis is one of the important respiratory diseases of sheep responsible for the low productivity due to economic losses resulting from death, reduced live weight, delayed marketing, treatment cost and un-thriftiness among survivors [6–8]. The term pasteurellosis was broadly used to designate a number of infections in domestic animals mainly caused by three species notably *M. haemolytica, B. trehalosi* and *P. multocida.* These bacterial species are non-motile, non-sporing, aerobic, fermentative, Gram negative rod and cocco-bacilli usually being pleomorphic [9, 10]. They can normally exist as commensals in the respiratory tract of cattle, sheep, and other ruminants [11–13].

The disease in sheep is characterized by an acute infection with high fever (40.4 °C to 42 °C), coughing, dyspnea, muco-purulent nasal discharge, anorexia and depression [14] that commonly develops when the immune system of the animal is compromised by stress factors such as crowding, transportation, draught, and inclement weather [15].

Pneumonic pasteurellosis is primarily caused by *M. haemolytica* [16] and is one of the major causes of mortality in domestic ruminants [17]. Apart from lung infection, *M. haemolytica* is also associated with systemic infections in small ruminants [17]. The taxon name *Mannheimia haemolytica* came into being after a number of works on reclassification and naming, first as *Bacterium bipolare multocidum* designated by Theodore kitt in1885 [18] and later in 1932 as *Pasteurella haemolytica* [18]. *P. haemolytica* was then classified into two biotypes viz. A and T based on its ability to ferment the sugars arabinose and trehalose, respectively [18]. These biotypes were further subdivided into thirteen A serotypes (A1, A2, A5, A6, A7, A8, A9, A11, A12, A13, A14, A16 and A17) and four T serotypes (serotypes 3, 4, 10 and 15) based on capsular antigen typing using Indirect Haemagglutination Test [19]. *Pasteurella haemolytica* biotype A was later allocated to a new genus *Mannheimia* and renamed as *Mannheimia haemolytica* while the 4 T serotypes were named *Bibersteinia trehalosi.* Recently, however, serotype A11 is classified into a new taxon as *M. glucosida* due to its different biochemical profile, leaving twelve serotypes of *M. heamolytica* [20].

Few studies have been conducted in northern, central highlands and eastern Ethiopia to determine the extent of the problem and the relative distribution of the different biotypes and serotypes of *M. haemolytica*. The studies indicated that ovine pasteurellosis is a major threat to sheep production and most serotypes of *M. haemolytica* biotype A are involved in causing pneumonic pasteurellosis with serotype A2 being the most prevalent [5, 20–23].

Since pneumonic pasteurellosis is one of the serious problems of small ruminants, effective control and prevention of the disease is mandatory. The traditional therapy based on the extensive use of antibiotics, including mass medication of animals, has caused an increase in the incidence of multi-drug resistant *M. haemolytica* strains in many parts of the world [24, 25]. Hence, an alternative prophylactic strategy through vaccination is more desirable.

In Ethiopia, a monovalent vaccine (inactivated *P. multocida* biotype A) produced at the National Veterinary Institute (Ethiopia) is being used for vaccination against ovine pasteurellosis although studies in central highlands of Ethiopia indicate *M. haemolytica* serotype A2 and A7 were reported to occur at high frequency [5, 26]. Although there is no published report on the efficacy of the currently used vaccine under field conditions, customer complaints on the high rates of mortality and morbidity following respiratory distress in different parts of the country have been documented despite the annual vaccination. This may suggest the need for the development of a multivalent vaccine using the most prevalent serotypes [5]. However, there is a need of an extensive study on identification of the prevalent serotypes in the country to be considered for inclusion into multivalent vaccine.

Despite the few reports on the serotypes of *M. haemolytica* circulating in Ethiopia, information on the genotypes of *M. haemolytica* associated with pneumonic cases in sheep in central Ethiopia is lacking which is essential for designing appropriate vaccine and molecular detection assays. Therefore, this study is aimed at identification and molecular characterization of *M. haemolytica* isolated from pneumonic cases of sheep in selected areas in central Ethiopia.

## Methods

### Study areas and animals

The study was conducted from November 2016 to May 2017 in four selected areas of central Ethiopia, which include Addis Ababa and three districts viz. Ada’a, Lome and Adama. All the study sites are located within the range of 100 km South East of Addis Ababa. Cases of sheep suffering from respiratory distress were purposively included in the present study for bacteriological analysis. Accordingly, pneumonic individual cases presented to veterinary clinics/veterinary health posts of the study districts/sites were considered for sampling. Since all cases were sampled from different farmers, it is assumed that each case originated from a different flock or herds.

### Sampling

All sheep were clinically examined and those with signs of anorexia, coughing, dyspnea, lethargy, serous to muco-purulent nasal discharge, and fever were considered for sampling. Accordingly, a total of 76 cases suspected of pneumonic pastuerellosis were included in the study comprising 11 from Addis Ababa, 27 from Ada’a, 17 from Lome and 21 from Adama.

After disinfection of external part of the nose with 70% alcohol, nasal swabs were collected from the nostrils using sterile cotton swabs as described previously by Carter [27–29]. The swabs were placed in labeled sterile test tubes that contain 2 mL of Amies transport medium (OXOID Hampshire, England) and was then kept in an ice box during transportation to the National Veterinary Institute.

### Bacteriological analysis

Isolation and identification of *M.haemolytica, B.trehalosi* and *P.multocida* was performed at bacteriology laboratory of the National Veterinary Institute as per the standard bacteriological techniques described previously [27, 28].

Nasal swabs were directly streaked onto blood agar base (Parck scientific limited, Northampton, UK) supplemented with 5% defibrinated sheep blood followed by incubation at 37 °C for 24–48 h.

In culture positive plates, typical suspected colonies (at least three colonies from a single plate) were picked and gram stained. Those colonies with gram reaction and colony morphology consistent to *Pasteurella species* were further sub-cultivated on blood and MacConkey agar plates (Himedia, India) to get pure cultures for further analysis. Pure cultures of single colony type from MacConkey agar was transferred onto nutrient agar (TSA) for a series of primary tests including tests for motility using mannitol motility medium, catalase (Fisher Chemical, UK), oxidase (Merck Co., Germany) and fermentative/oxidative tests using OF Basal Medium (Titan Biotech Ltd., India) as described in Quinn et al. [28, 29].

Presumptive species identification of isolates was done employing secondary biochemical tests which included: tests for urease activity, fermentation of sugars such as glucose, sucrose, lactose and tests for production of indole following standard procedures described previously [29, 30].

### Molecular identification and characterization

#### Deoxyribose nucleic acid (DNA) extraction

Few colonies from the presumptively identified 24–48 h pure cultures of *M. haemolytica* and *B.trehalosi* were picked for DNA extraction using Qiagen DNeasy Blood and Tissue Kit as per the manufacturer’s instructions (Qiagen, Germantown, MD, USA).

#### Multiplex PCR assay for detection of virulence associated genes of M. haemolytica

Primers targeting virulence associated genes of *M. haemolytica* viz. *PHSSA* (*Pasteurella haemolytica* serotype specific antigen) gene coding for *M. haemolytica* serotype specific antigen and *Rpt2* gene loci coding for methyltransferase (Table 1) were used in multiplex PCR (mPCR) assay described in previous studies [31]. In brief, the PCR was carried out in a final volume of 25 μl of reaction mixture containing 10 μl of IQ Super mix (Bio Rad, USA) (DNA polymerase, dNTPs and buffer), 2 μl (5pM/μl) of each primer pairs, 3 μl of RNase free water and 4 μl template DNA. The PCR condition used was an initial denaturation at 95 °C for 3 min, followed by 35 cycles of each at 95 °C for 1 min, annealing at 48 °C for 1 min and extension at 72 °C for 30s and a final extension cycle at 72 °C for 5 min. One reaction tube without the DNA template and the other with DNA template from reference *M. haemolytica* isolate from National Veterinary Institute culture collection (MH-NVI) were included as negative and positive controls, respectively.

**Table 1:** Primer pairs flanking capsular genes of *P. multocida* and virulence associated genes of *M. haemolytica* used in the current study

Gene locus	Primer sequence (5′- 3′)		Size (bp)	Reference
*CapA*	Forward	5’-TGCCAAAATCGCAGTCAG- 3’	1044	[32]
	Reverse	5′ -TTGCCATCATTGTCAGTG- 3’		
*PHSSA*	Forward	5′ -TTC ACA TCT TCA TCC TC-3’	325	[31]
	Reverse	5′ TTT TCA TCC TCT TCG TC-3’		
*Rpt2*	Forward	5′ - GTT TGT AAG ATA TCC CAT TT- 3’	1022	[31]
	Reverse	5′- CGT TTT CCA CTT GCG TGA − 3’		

#### PCR for the detection of *P. multocida*

Isolates presumptively identified as *M.haemolytica* and *B.trehalosi* were subjected to PCR assay using specific primer pairs targeting the capsular biosynthesis gene (*capA*) (Table 1) of *P. multocida* as described previously by Townsend et al [32].

PCR reaction mixture (50 μl) containing, master mix (Fermentas, Thermo Fisher Scientific, USA), 6 μL of 5 pmol of each primer (Eurofins MWG Operon, Germany), 6 μL DNA template and 20 μL IQ super mix were used. The amplification protocol used was an initial denaturation at 95 °C for 5 min, followed by 35 cycles each at 95 °C for 1 min, annealing at 55 °C for 1 min and extension at 72 °C for 30 s and a final extension cycle at 72 °C for 7 min. Negative and positive controls were included one without DNA template and the other reaction tube with DNA from *P. multocida* strain from NVI collection (MH-NVI), respectively. Detection of the PCR products was done in 2% (*w*/*v*) agarose gel, prepared from 0.5X Tris borate EDTA buffer stained with Gelred. Each PCR product (5 μl) was mixed with 6X loading buffer and loaded into separate well of the pre-prepared gel while 1 kb plus DNA molecular marker was loaded onto the first and last lane and run at 120 V for 60 min on electrophoresis apparatus (EC 2060, USA). The different band sizes of the PCR products were visualized under UV transilluminator and photographed in gel documentation system (UVI TEC, UK).

#### Purification and sequencing of PCR products

The PCR products were purified using the Wizard SV Gel and PCR clean-up system kit (Promega, Germany) and its concentration determined using the NanoDrop 2000c spectrometer (Thermo Scientific, USA). The concentration of each purified product was adjusted and prepared according to the instruction recommended by the sequencing company and submitted for sequencing along with sequencing primers (forward and reverse) to LGC Genomics (Berlin, Germany).

### Phylogenetic analysis or genotyping

Genotyping of the isolates was conducted using partial sequence data of two virulence genes, *PHSSA* and *Rpt2* genes. Since the current isolates were from the same region i.e. central Ethiopia, genetic analysis using virulence genes is expected to provide better resolution as they are known to be under high evolutionary pressure. *PHSSA* and *Rpt2* gene sequences of *M.haemolytica* strains isolated in the current study and the accession numbers of the sequences retrieved from GenBank database used in the current analysis is presented in Table 2.

**Table 2:** Nucleotide accession numbers of *PHSSA* and *Rpt2* gene sequences of the current isolates and reference strains *M. haemolytica* included in the analysis

Isolate	Accession no^a^	Source or reference
*M. haemolytica* strain ETH/Adama/21/2017	MH220354*	This study
*M. haemolytica* strain ETH/Ejere/16/2017	MH220353*; MH220358**	This study
*M. haemolytica* strain ETH/Ejere/15/2017	MH220355*; MH220359**	This study
*M. haemolytica* strain ETH-DZ/kality 26/01/2017	MH220357**	This study
*M. haemolytica* strain ETH-Dalota-08/12/2016	MH220360**	This study
*M. haemolytica* NVI-reference strain	MH220356**	This study
*M. haemolytica* strain USDA-ARS-USMARC-185	CP004753.2	[38]
*M. haemolytica* strain M42548	CP005383.1	[38]
*M. haemolytica* strain D174	CP006574.1	[39]
*M. haemolytica* strain D171	CP006573.1	[39]
*M. haemolytica* strain D153	CP005972.1	*.* [39]
*M. haemolytica* strain CSWRI/AH/12/12	KJ534629.1	[31]
*M. haemolytica* strain CSWRI/AH/11/12	KJ566123.1	[31]
*M. haemolytica* strain 89,010,807 N	CP011098.1	[40]
*M. haemolytica* strain 193	CP023043.1	[40]
*M. haemolytica* strain 191	CP023044.1	[40]
*M. haemolytica* USDA-ARS-USMARC-184	CP006957.2	[38]
*M.haemolytica* strain 89,010,807 N lktA	CP011099.1	[40]
*M. haemolytica* USDA-ARS-USMARC-183	CP004752.2	[38]
*M. haemolytica* strain 187	CP023046.1	[38]
*M. haemolytica* strain 186	CP023047.1	[38]
*M. haemolytica* strain CSWRI/AH/MhA.16	MF417618.1	[41]
*M. haemolytica* strain 30/30–02-16Lc	MF776879.1	[41]
*M. haemolytica* serotype A1	AF060119.2	[42]

^a^Acc.no refer to both *PHSSA* and *Rpt2* gene sequences; *Acc.no for only *PHSSA* gene; **acc.no for only *Rpt2* gene

### Data analysis

Descriptive statistics were used in summarizing quantitative data when appropriate. Trace sequence data were edited and fragments were assembled using Vector NTI Advance™ 11.5 software (Invitrogen, Carlsbad, CA, USA). A consensus sequence was generated for each isolate from forward and reverse sequences using BioEdit. For comparative phylogenetic analysis, blastn was used to collect additional sequence data of *PHSSA* and *Rpt2* genes from reference *M. haemolytica* strains and isolates from other geographical areas from GenBank. All sequence analysis was conducted in MEGA version 7. Multiple sequence alignments were performed using Clustal W [33]. Phylogenetic relationships among the current isolates and isolates from other geographical areas was determined based on phylogenetic trees constructed using the Neighbor-Joining algorithm with interior-branch test and the complete deletion option with bootstrap replicates set at 1000.

## Results

*M. haemolytica* was found to be commonly associated with pneumonic cases of sheep in the study areas where it was isolated from 26 of the total 76 (34.21) cases while *Bibersteinia trehalosi* was associated with two of the cases (Table 3) although the majority of the cases were due to some other agents that need yet to be determined. The biochemical profile of the isolates presumptively identified as *M. haemolytica* and *B. trehalosi* is presented in Table 4.

**Table 3:** Recovery rate of isolates with respect to the study areas

Study sites	Number tested	Isolates		Total *#* (%)
		*M.haemolytica*	*B.trehalosi*	
		# (%)	# (%)	
Addis Ababa	11	2 (18.18)	nil	2 (18.18)
Ada’a (DZ/Kality)	27	10 (37.03)	2 (7.41)	12 (44.44)
Lome (Ejere)	17	8 (47.06)	nil	8 (47.06)
Adama	21	6 (28.57)	nil	6 (28.57)
Total	76	26 (34.21)	2 (2.63)	28 (36.84)

**Table 4:** Biochemical characteristics of the isolates presumptively identified as *M. haemolytica* and *B. trehalosi*

Reaction	*Isolates (No = 26)*	*Isolates (No = 2)*
Haemolysis	+	+
Motility	–	–
Catalase	+	+
Oxidase	+	+
Growth on MacConkey	+	+
Indole production	–	–
Glucose	+	+
Lactose	+	–
Sucrose	+	+
Interpretation	*M. haemolytica*	*B. trehalosi*

**Fig. 1:**
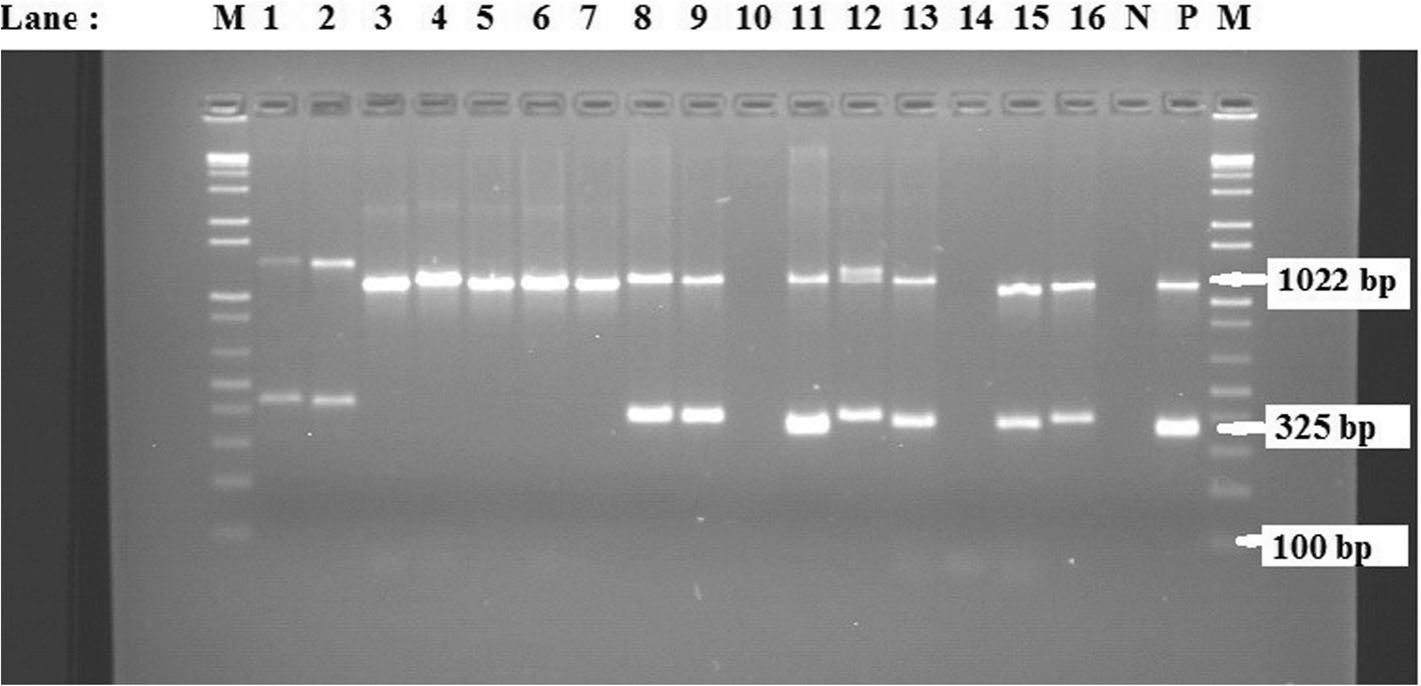
Agarose gel electrophoresis showing PCR products of *Rpt2* and *PHSSA* genes approximately 1022 and 325 bp, respectively. Lanes: M = 1 kb plus DNA molecular markers; 1–16: Isolates (lane 10 and 14: *B.trehalosi* isolates, please describe the other *M. haemolytica* positive lanes). N: Negative control-; P: positive control

Further molecular analyses of the isolates using primers targeting *PHSSA* and *Rpt2* genes of *M. haemolytica* in a multiplex PCR assay resulted that all 26 isolates phenotypically identified as *M. haemolytica* were positive for *Rpt2* gene, 21 of them being positive for *PHSSA* gene thus belonging to serotype A1. Nine representative *M. haemolytica* isolates positive for both *Rpt2* and *PHSSA* genes while 5 representatives positive only for *Rpt2* gene are shown in Fig. 1.

**Fig. 2:**
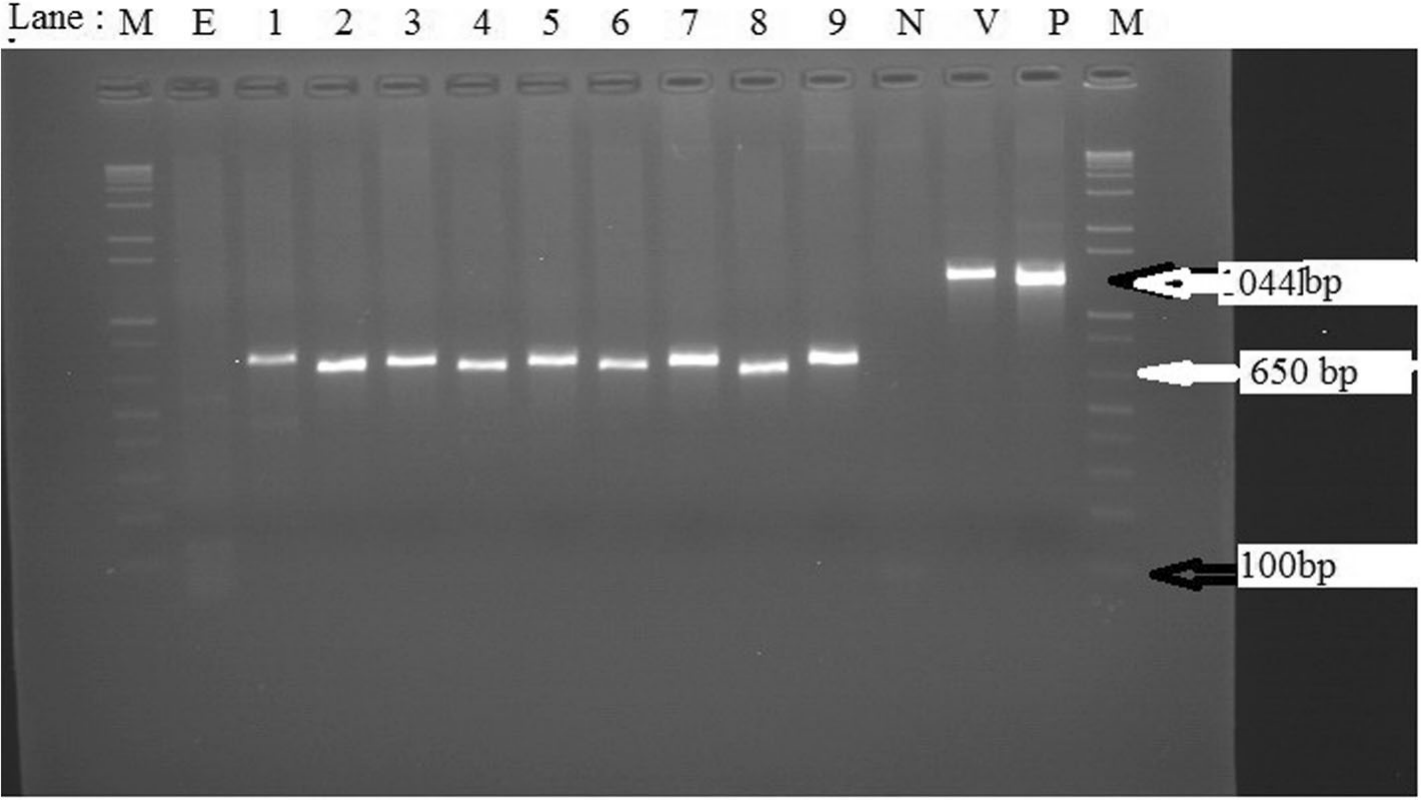
Agarose gel electrophoresis showing PCR products (approximately 1044 bp) using primer pairs targeting capsular biosynthesis gene of *P. multocida*. Lanes: M = 1 kb plus DNA molecular marker, E: Extraction control, Lane 1–9:*M.haemolytica* isolates. N: Negative control, V: NVI vaccine strain (*P. multocida type A*) positive around 1044 bp. P: positive control

**Fig. 3:**
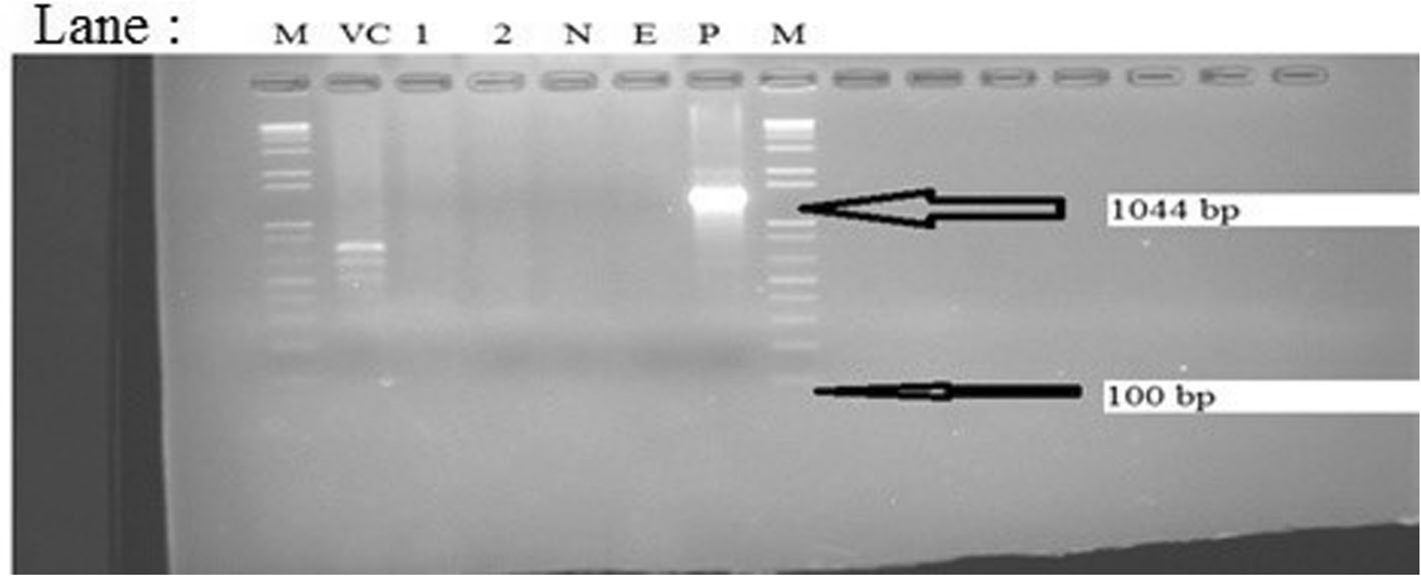
Agarose gel electrophoresis showing PCR products (approximately 1044 bp) using primer pairs targeting capsular biosynthesis gene of *P. multocida* and *B trehalosi* as template. Lanes: M = 1 kb plus DNA molecular marker, Lane VC: NVI reference strain (*M. haemolytica* serotype A2 isolate), Lane 1–2: *B. trehalosi* isolates, N: Negative control; E: extraction control, P: positive control

When analysed in a PCR assay using primers specific for capsular biosynthesis gene of *P. multocida, M. haemolytica* isolates included were negative resulting in rather non-specific PCR product size of about 650 bp different from the expected 1044 bp (Fig. 2). Both isolates phenotypically identified as *B. trehalosi* were also negative for presence of both *PHSSA* and *Rpt2* genes of *M. haemolytica* as well as for capsular gene of *P. multocida* (Fig. 3).

### Phylogenetic analysis

**Fig. 4:**
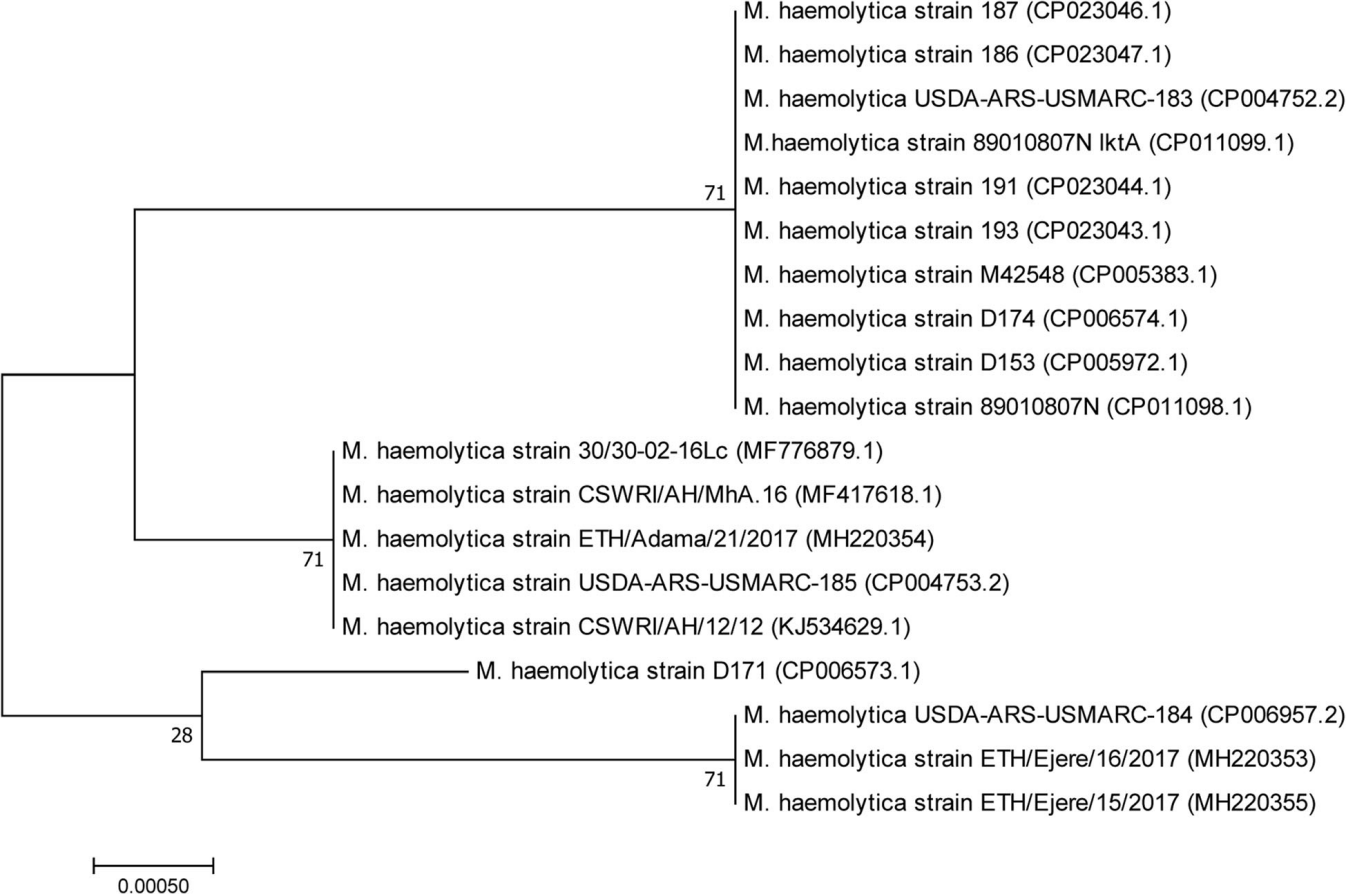
Evolutionary relationships of 19 *Mannheimia haemolytica* isolates including three isolates from Ethiopia with respect to *PHSSA* gene partial sequence. Codon positions included were 1st + 2nd + 3rd + Noncoding. All positions containing gaps and missing data were eliminated. There were a total of 301 positions in the final dataset

Phylogenetic analysis based on *PHSSA* gene showed that *M. haemolytica* isolates from Ethiopia resolved into two genotypes consistent to the site of isolation, an isolate from Adama (ETH/Adama/21/2017) being phylogenetically related to *M. haemolytica* strains from India (CSWRI/AH/12/12; CSWRI/AH/MhA.16 and 30/30–02-16Lc) and USA (USDA/ARS/SAM/185) while two of the isolates from Ejere, Ethiopia formed distinct cluster (ETH/Ejere 15/2017 and ETH/Ejere 16/2017) with another strain from USA (USDA/ARS/USMARC/184) (Fig. 4).

**Fig. 5:**
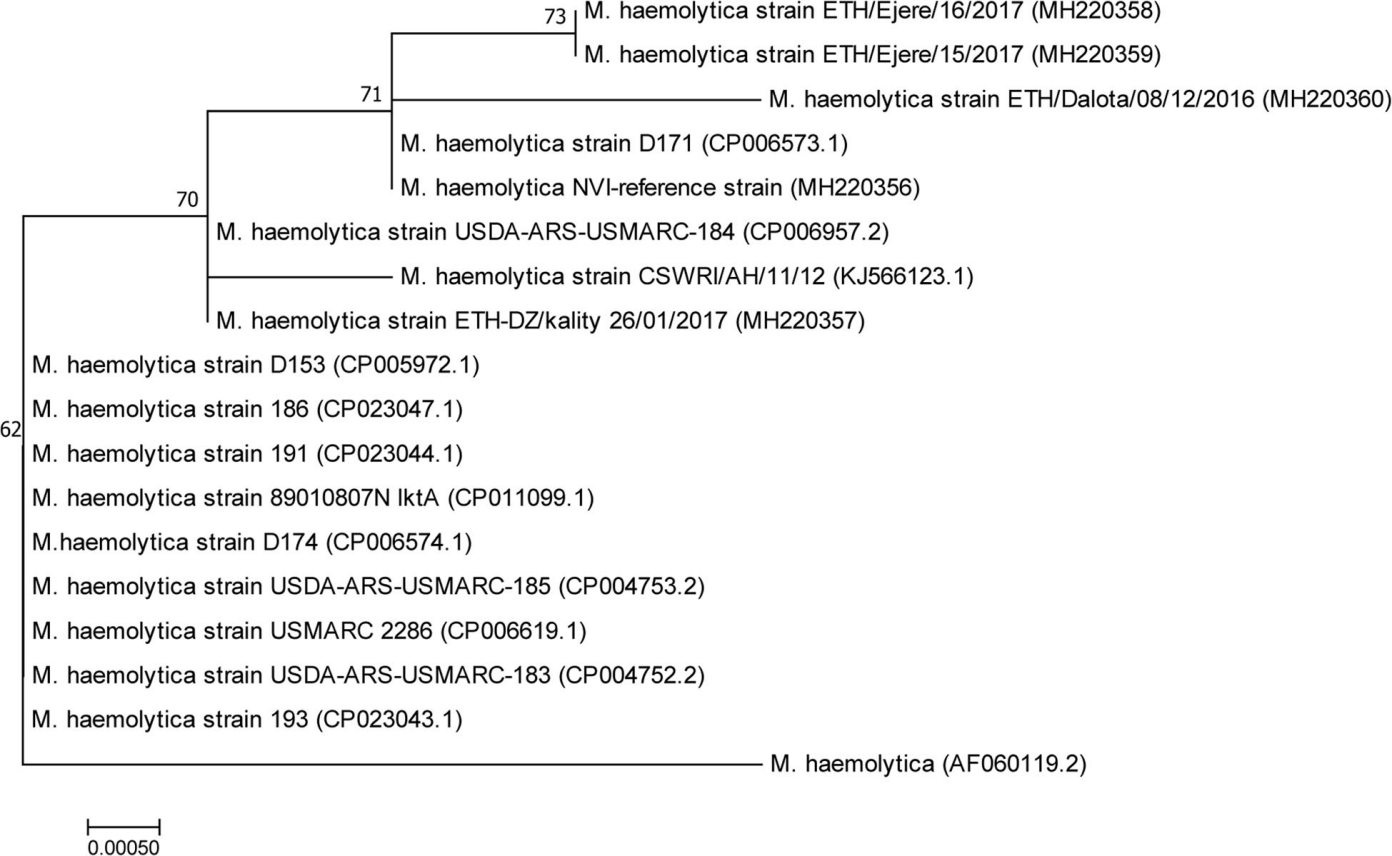
Phylogenetic analysis of 18 *Mannheimia haemolytica* strains including four isolates from Ethiopia using *Rpt2* gene sequence. Codon positions included were 1st + 2nd + 3rd + Noncoding. All positions containing gaps and missing data were eliminated. There were a total of 780 positions in the final dataset

On the other hand, phylogenetic analysis using *Rpt2* gene showed similar scenario where all isolates from the study sites were resolved into three genotypes which is consistent to the site of isolation. Two of the isolates from the same site (ETH-Ejere-16/2017 and ETH-Ejere-15/2017) which were analysed in both genes seem to be clonal complexes forming distinct cluster. The other two isolates (ETH-Dalota-08/12/2016 and ETH-DZ/kality 26/01/2017) seem to represent distinct genotypes although strain from Dalota is closer to the ETH-Ejere clonal complex. However, the strain from ETH-DZ/kality is genetically far related to the rest of the strains from Ethiopia and any of the reference strains included in the analysis. Moreover, based on the *Rpt2* gene sequence analysis all the three genotypes of *M. haemolytica* identified were far related to the *M. haemolytica* NVI reference strain (Fig. 5).

## Discussion

The present study demonstrated that *M.haemolytica* was associated with one third of pneumonic cases indicating its significant role in pneumonia of sheep in central Ethiopia although other agents associated with the remaining cases need yet to be determined. This study is the first of its kind that revealed the genotypes of *M. heamolytica* circulating among cases of pneumonic sheep in central Ethiopia.

The clinical findings observed in affected sheep were consistent with the clinical pictures associated with *M. haemolytica* and *B.trehalosi* infections as reported previously [14, 34, 35]. The absence of *P. multocida* from any of the cases investigated in contrast to *M. haemolytica* indicate the limited role of *P. multocida* in causing pneumonic pasteurellosis in central Ethiopia unlike the previous reports. The study rather indicated that *B. trehalosi* was associated with few of pneumonic cases of sheep (7.14%) which may suggest its potential role as cause of pneumonia in the study areas although it was quite lower than previous reports of 13.4% [20] from south Wollo, Northern Ethiopia. Similar occurrence of *B. trehalosi* was obtained from central Ethiopia by Mekonnen [21] which was 7%. The absence of *P.multocida* in the current study is in agreement with previous report in which *P.multocida* was not isolated from cases of pneumonic sheep [36].

Phenotypic methods used in the current study couldn’t distinguish *M. haemolytica* from *B.trehalosi* except for lactose fermentation which is consistent with Korczak et al. [37] who stated that *B. trehalosi* is phenotypically closely related to *Mannheimia*. All isolates phenotypically identified as *M. haemolytica* were also confirmed to be as such by the mPCR assay indicating that the standard phenotypic method can provide reliable identification in the absence of molecular technique.

The non-specific PCR product obtained in PCR assay targeting capsular biosynthesis gene of *P. multocida* when applied to *M. haemolytica* isolates may show that *P. multocida* and *M.haemolytica* share common sequences in their capsular gene(s) at the primer binding site but at different positions. Isolates phenotypically identified as *B.trehalosi* were not detected by any of species specific primers for *P. multocida* and virulence associated genes *(PHSSA and Rpt2)* of *M.haemolytica* showing their genetic distinction, which is also corroborated by previous studies with respect to *rpoB* gene [37]. *M. haemolytica* and *B. trehalosi* belong to different distinct clusters in phylogenetic analysis using the *rpo*B gene, a gene encoding for the β subunit of bacterial RNA polymerase [37]. The association of *M. haemolytica* with significant proportion of ovine pneumonic cases in the current study is in line with those mentioned by Sisay and Zerihun [26], Ayelet et al. [5], and Deressa et al. [22] in central highlands and Eastern Ethiopia where all indicated *M. haemolytica* as the main culprit of the problem. Thus, the current finding along with previous reports of the high prevalence of *M. haemolytica* serotype A2 and A8 in other parts of central Ethiopia [5] all consolidate the need for polyvalent vaccine unlike the currently used monovalent vaccine (inactivated *P. multocida* biotype A). The high rates of mortality and morbidity following respiratory distress in different parts of the country despite the annual vaccination may indicate the reduced efficacy of the currently used vaccine supporting our findings. However, assessment of outbreaks at national level to determine the role of *M. haemolytica* and other agents involved in causing pneumonia in sheep and further molecular identification of the circulating *M. haemolytica* serotypes is essential to have an overview on major causative agent and serotype distribution for selection of candidate vaccine strains that can provide cross protection.

The existence of three genotypes of *M. haemolytica* circulating in the study areas based on the results of phylogenetic analysis with respect to partial sequences of *Rpt2* and *PHSSA* genes indicate the involvement of different strains of varying sources causing outbreaks. Except the *M. haemolytica* isolates from Lome Ejere, which seem to be clonal complexes, the rest of the isolates analysed belonged to diverse genotypes different from the reference local strain from NVI. This variation in genotype distribution among *M.haemolytica* isolates observed is in agreement with the findings of Gilmour [12] who stated that members of the genus *Mannheimia* are phenotypically and genotypically heterogeneous. This suggests that further nation-wide strain characterization is important with subsequent work on whether these different genotypes share common antigens which is vital for an effective vaccine development. The fact that *M. heamolytica* was found to be associated with one third of sheep pneumonic cases in the current study highlights the role of one or more or a combination of other agents in causing sheep pneumonia in the majority of the cases requiring further studies targeting all possible agents.

## Conclusion

Both phenotypic and molecular characterization confirmed that *M. haemolytica* is associated with a significant number of pneumonic cases of sheep in study areas of central Ethiopia although the role of other agents responsible for the majority of the cases is yet to be determined. Phylogenetic analysis based on partial sequences of *PHSSA* and *Rpt2* gene of the current isolates revealed the existence of three genotypes of *M. haemolytica* circulating in the study areas, which was consistent with the site of isolation. Further extensive work to figure out strain (genotype) distribution, antigenic relationships among strains, understanding the molecular epidemiology of *M.haemolytica* and *B.trehalosi* at national level and other important agents/factors to the disease has paramount importance in designing a cost effective prevention and control method.

## Abbreviations

DNA: Deoxyribose nucleic acid
mPCR: Multiplex Polymerase Chain Reaction
NVI: National Veterinary Institute
*PHSSA*: *Pasteurella haemolytica* serotype specific antigens
